# Patient Safety Event Reporting and Opportunities for Emergency Medicine Resident Education

**DOI:** 10.5811/westjem.2020.3.46018

**Published:** 2020-06-15

**Authors:** V. Ramana Feeser, Anne Jackson, Regina Senn, Timothy Layng, Sally A. Santen, Angela B. Creditt, Harinder S. Dhindsa, Michael J. Vitto, Nastassia M. Savage, Robin R. Hemphill

**Affiliations:** * Virginia Commonwealth University School of Medicine, Department of Emergency Medicine, Richmond, Virginia; † Virginia Commonwealth University Health System, Department of Emergency Medicine, Richmond, Virginia; ‡ Virginia Commonwealth University Health System, Department of Emergency Medicine, Division of Emergency Medical Services, Richmond, Virginia; § Virginia Commonwealth University School of Medicine, Office of Assessment, Evaluation, and Scholarship, Richmond, Virginia

## Abstract

**Introduction:**

Healthcare systems often expose patients to significant, preventable harm causing an estimated 44,000 to 98,000 deaths or more annually. This has propelled patient safety to the forefront, with reporting systems allowing for the review of local events to determine their root causes. As residents engage in a substantial amount of patient care in academic emergency departments, it is critical to use these safety event reports for resident-focused interventions and educational initiatives. This study analyzes reports from the Virginia Commonwealth University Health System to understand how the reports are categorized and how it relates to opportunities for resident education.

**Methods:**

Identifying categories from the literature, three subject matter experts (attending physician, nursing director, registered nurse) categorized an initial 20 reports to resolve category gaps and then 100 reports to determine inter-rater reliability. Given sufficient agreement, the remaining 400 reports were coded individually for type of event and education among other categories.

**Results:**

After reviewing 513 events, we found that the most common event types were issues related to staff and resident training (25%) and communication (18%), with 31% requiring no education, 46% requiring directed educational feedback to an individual or group, 20% requiring education through monthly safety updates or meetings, 3% requiring urgent communication by email or in-person, and <1% requiring simulation.

**Conclusion:**

Twenty years after the publication of *To Err is Human*, gains have been made integrating quality assurance and patient safety within medical education and hospital systems, but there remains extensive work to be done. Through a review and analysis of our patient safety event reporting system, we were able to gain a better understanding of the events that are submitted, including the types of events and their severity, and how these relate to the types of educational interventions provided (eg, feedback, simulation). We also determined that these events can help inform resident education and learning using various types of education. Additionally, incorporating residents in the review process, such as through root cause analyses, can provide residents with high-quality, engaging learning opportunities and useful, lifelong skills, which is invaluable to our learners and future physicians.

## INTRODUCTION

Healthcare systems often expose patients to significant, preventable harm on par with other chronic medical conditions at rates estimated between 44,000 and 98,000 deaths annually,^1^ although some suggest it may be even higher.^2^ These reports have highlighted the importance of patient safety and safety event reporting. These reporting systems allow for local review of events and identification of whether they are local issues or a system-level vulnerability.^3^,^4^ Aligned with efforts to identify such errors, research is beginning to focus on how we learn from the reported events. One benefit of reporting is the potential to reprioritize, learn, and fix system processes by identifying contributing factors and helping providers address these issues.^5^–^9^ However, research has been limited. Improvements have been made in incident reporting, but this alone does not lead to systems changes or enhanced patient care. Pronovost et al concluded that reporting systems alone were “insufficient to gain the knowledge needed to learn how [patient safety report systems] can improve patient safety.”^10^,^11^ It must also include the establishment of organizational leadership and safety champions to spearhead learning from events.^12^

While health systems have reporting structures and processes to address patient safety, residents are not always purposefully engaged in reporting problems and vulnerabilities. The Accreditation Council for Graduate Medical Education (ACGME) through the Clinical Learning Environment Review and milestone competencies has increasingly stressed resident education about safety events, along with other important educational domains. However, it is unclear how safety event reporting has been used to educate residents. Within patient safety reporting systems, residents have the opportunity to be actively engaged in the identification of adverse events, near misses, unsafe conditions, and potential systems issues. However, to successfully instill lifelong, improvement-based practices within our physician learners, we must close the loop by providing feedback, education, and enhanced training opportunities based on submitted safety reports, including those by residents and others.

As a high percentage of care in an academic emergency department (ED) is provided by residents, it is critical to include them in interventions and educational initiatives to address patient safety in the ED. From our own institutional resident learning environment survey, ED residents indicated that they received adequate feedback on safety event reports submitted through the formal submission system only 50% of the time. This finding suggested that resident input into safety may not be well considered, encouraging a deeper look into the use of our safety event reporting system. This low rate of feedback is concerning because residents appear to be trying to engage in the safety reporting system, but the lack of feedback may discourage their engagement in safety event reporting. Therefore, the objectives of this study were first to analyze patient safety reporting and, second, determine the urgency and opportunity for resident learning and education from the event report.

**Population Health Research Capsule**

What do we already know about this issue?

*Academic EDs are tasked with educating residents while still providing high quality care. When errors occur, residents often do not receive adequate feedback*.

What was the research question?

How can patient safety event reports identify opportunities for EM resident education and interventions?

What was the major finding of the study?

*69% of event reports required educational intervention with 46% needing individual/group directed feedback*.

How does this improve population health?

*By using safety event reports to inform and educate, residents can know how to help correct identified system errors to prevent further unsafe events*.

## METHODS

### Setting

The included patient safety event reports for this study are from an inner city ED from the Virginia Commonwealth University Health System with approximately 100,000 visits annually. All providers and staff are encouraged to enter patient safety net reports (PSN) into an online system. Residents are encouraged to submit one to two PSNs a year. The standard process is that PSNs are reviewed and addressed by the ED quality and safety leadership team, which includes an attending physician, the nursing director, and a registered nurse.

### Coding

To meet the first objective of understanding and categorizing the types of PSNs, the team determined categories for the PSNs based on the literature and the expertise of the research group (Table 1). Then three team members, subject matter experts who routinely review and address PSNs (i.e., attending physician, nursing director, and registered nurse), categorized 20 PSNs together and resolved any issues or gaps identified in the coding schemas. They then coded 100 safety reports individually to determine the level of inter-rater reliability using kappa, which indicated high levels of agreement (0.92). The high inter-rater reliability indicated that they could reliably code the remaining 400 safety reports with a single coder.

The PSNs were coded in multiple categories (Table 1). We looked specifically at the types of events and how they should be addressed through resident and staff education. The types of education were coded to identify how best to respond to safety event reports such that the residents would benefit from their submission and resolution. The events that could result from safety event reporting were categorized into the following five levels: 0 – no education; 1 – directed feedback to individual/group; 2 – quarterly/monthly educational update; 3 – urgent communication (e.g., email within one week; discussion at resident conference, daily huddles, or morbidity and mortality presentation [M&M]); and 4 – provider simulation.

The criticality of the event was determined by the ED quality and safety leadership team. If the event was immediately life threatening, it was deemed of critical importance. If the report focused on something that needed to be addressed but was not of immediate importance, it was categorized as an actionable event that allowed time to research the most effective way to address the event. To code whether the events in the PSNs were addressed in the moment, the coders reviewed the event description provided in the submission. If the event description included details of the event having been addressed at the time of the occurrence, then it was coded as having been addressed, whereas if the description was clearly indicating the event was not addressed, it was coded as such. Those PSNs that did not provide sufficient detail were coded as being unknown whether it was addressed at the time of the event.

## RESULTS

From January 1, 2019, to May 31, 2019, 513 PSNs were submitted for the ED. Of these, 4.5% (23) caused harm including two deaths. (It was not clear whether the patients died directly from the event.) An additional 18% (92) of patients required additional treatment related to the event, 21% (108) reached the patient in some way (e.g., inconvenience, inefficiency, redundant tests), and the remaining 56% (288 patients) were near misses – unsafe events that resulted in no harm to patients.

All PSNs were also categorized by the type of action that should be taken in response to the reported safety event. Of the 513 PSNs, 2% (10) required a critical review or action, which includes the ED quality committee (i.e., an attending physician, the nursing director, and a registered nurse) reviewing the event, conducting a root cause analysis, and addressing the systems issues (e.g., communication and team breakdown; failure to rescue or escalate; vulnerabilities within the informatics system; etc); 78% (400) were actionable but did not require critical action (e.g., direct communication to residents or group communication about systems or equipment); and 20% (103) were not actionable (i.e., based on the PSN, no further action was required). The majority of the PSNs (79%, 405 events) were addressed in the moment when the event happened compared to 3% (17) in which the reporter or team did not address the issue at the time. However, 19% (17) of PSNs contained insufficient information as to whether the event was addressed in the moment and were coded as “unknown.”

There were many different foci for the PSNs (Table 2), including some submissions that had multiple foci (e.g., employee behaviors, patient assessment issue, a policy/procedure issue, and nursing documentation issue). However, the most common events were issues related to staff and resident training (25%, 129); communication (18%, 93); and equipment (14%, 71). The PSNs were then categorized to what type of action should be taken based on the event.

### Approach to Safety Event Education

The type of educational intervention that should have been used was determined within the department based upon the type and severity of event. These interventions could be provided through the traditional venues for communication built into residency programs, such as M&Ms, conferences, and mentoring relationships for one-on-one developmental feedback. The relevance of the safety events to residents was determined by whether a resident was directly engaged in the event if known and the potential value to residents’ long-term capabilities if education related to the event were provided. This value was determined by the ED quality and safety leadership team review in collaboration with the ED residency leadership team to determine what appropriate educational opportunities related to the safety event reports would be.

About one third (31%) of PSNs required no educational intervention. Nearly half of the PSNs (46%, 235) were educational level 1 that would require directed educational feedback to an individual or group. Examples include a need to escalate to the attending for consult or admissions with a dialysis patient requiring bilevel positive airway pressure; delayed acceptance to the intensive care unit (ICU); and a delayed ultrasound to rule out deep vein thrombosis to be performed before transfer to an inpatient bed but there was no technician to perform inpatient studies overnight. As a result, educational feedback could be given to ED residents for alternative methods of dealing with similar situations, including escalation procedures when dealing with an interprofessional care team.

Twenty percent (100) of the PSNs were classified as level 2, indicating that education should be carried out through monthly safety updates or at faculty/resident meeting. Examples included the following: a long length of stay in the ED with patient decompensation that required escalation of care; a patient with a dangerous level of hyperkalemia and severe hyperglycemia who received calcium, bicarbonate, and albuterol but did not have an insulin drip started before transferring to the ICU; and a pediatric emergency physician-ordered medication based on the body mass index instead of the patient’s weight, resulting in improper dosage. Level 2 PSNs should result in a review of the incidents and the situational factors contributing to the events during monthly faculty and resident meeting and inclusion in safety updates.

Three percent (15) of PSNs were classified as a level 3, requiring urgent communication by email or in person at a meeting such as at a weekly resident conference. Examples of level 3 education included incorrectly discharging a cancer patient with hypercalcemia who required admission; inadequate antibiotic administration for aspiration pneumonia; and a misdiagnosis of gout, requiring subsequent admission for foot cellulitis that required surgical debridement. These events should result in immediate communication with the involved parties as well as an in-person debriefing to go over the specifics of the event.

Only three (<1%) reports (two of these reported the same event) were classified at the highest level 4 requiring simulation for providers. One was a pediatric death after ED discharge with a missed diagnosis, and the other was a retained guidewire during femoral central line placement. These could result in simulations related to the missed diagnosis or multiple practices placing central lines to ensure guidewires are removed. The remaining 30% of PSNs were determined to require no educational action.

## DISCUSSION

Twenty years have passed since the publication of *To Err is Human: Building a Safer Health System*, and while gains have been made, extensive work remains to be done. For instance, the ACGME has begun to require that programs include quality and safety training as part of resident education, stating: “Residents must demonstrate the ability to analyze the care they provide, understand their roles within health care teams, and play an active role in system improvement processes… to critique their future unsupervised practice and effect quality improvement measures.”^13^ To effectively provide feedback for PSNs, a method or algorithm must be developed so that every PSN submitted receives a meaningful response. Based upon our data and analyses, one method may be analyzing and categorizing the event type in the report and distinguishing the level of education required. Afterward, to ensure closed-loop communication regarding the submitted report, emails would be sent to the submitter (if identified) and those involved in the event (if any indicated) to inform them of the action plan and resolution of the report.

Our study identified categories that allow for key personnel and departments to easily track events, including the degree of harm and their frequency. This allows the quality team to more efficiently target those events that result in the most harm and for consideration of events that have lower level of harm potential but still occur more frequently. For instance, these may be events that are waiting for the right time to trigger a more significant event or they may be simple irritants that create sustained frustration. Identification of these types of events can be intriguing points of discussion for residents and provides them the opportunities to practice functional problem-solving skills with these smaller but frequent events prior to involvement in other more systemic and severe issues. Although it was not possible to determine which reports involved or were submitted by residents specifically, each provides an opportunity to impact resident education and the quality of care they provide.

Once safety events have been categorized and prioritized, it is much more feasible to consider the event’s specifics to determine the next steps to educate, improve, and prevent reoccurrence. However, depending on the event, it may only require direct communication and feedback to the individuals involved. Alternatively, review of the events in resident didactics, monthly safety newsletters, or simulations would be the preferred educational modality for more generalizable events and to provide education related to the event to all residents. Furthermore, incorporating feedback at an individual and resident level reinforces to the residents and other members of the department that submitted reports are taken seriously and are valued by the department. This may also encourage further participation in event reporting and, potentially, in quality improvement efforts (e.g., developing action plans or simulations around existing concerns). The inclusion of residents in department quality and safety committees provides residents with useful, lifelong skills, making such an experience invaluable.

## LIMITATIONS

There are several limitations to the results of this study. First, this was a single-institution, retrospective study, and the categories of safety events may need to be broadened or altered for other organizations and for prospective research efforts. Further, the safety event reporting system is for all members of the health system and does not require the submitters to include their role in order to encourage reporting and ensure confidentiality should there be any concerns of potential repercussions. Additionally, this reporting system does not allow an automatic pull of the role of the person that submitted the report, and this would have required each report to be opened manually to get this information. Thus, the events placed into this system include events beyond those submitted only by residents. This may have resulted in some events that had less specific relevance to resident concerns; however, those reports more focused on systems-issues still benefit residents as they should have the opportunity to learn how best to deal with those situations while still in an educational setting.

## CONCLUSION

Through systematic analysis and categorization of safety event reports, this study showed that these events can be used to develop specific learning tools. However, naturally, there are barriers to this process. Providing education and feedback to residents and other providers requires a great deal of time and manpower. Additionally, flaws within reporting systems themselves will continue to be discovered and require potential redesigns of the system overall or smaller changes intermittently. This necessitates supportive communication and a good working relationship between the department and health system. Regardless, the end result is worth the effort to implement this resident education-based system, given that “feedback and experiential learning are essential to developing true competence in the ability to identify causes and institute sustainable systems-based changes to ameliorate patient safety vulnerabilities.”^13^

## Figures and Tables

**Table 1:** Categorizing patient safety notes as part of process to determine how best to address concerns.

Category	Labels	Frequency (%)
Harm score		
	Unsafe condition	65 (13%)
	Near miss	97 (19%)
	No harm evident, physical or otherwise	126 (25%)
	Emotional distress or inconvenience	110 (21%)
	Additional treatment	92 (18%)
	Temporary harm	17 (3%)
	Permanent harm	4 (1%)
	Severe permanent harm	0 (0%)
	Death	2 (<1%)
Actionable		
	Critical action	10 (2%)
	Actionable	400 (78%)
	Not actionable	103 (20%)
Addressed in the moment		
	Yes	405 (79%)
	No	91 (18%)
	Unknown	17 (3%)
Target of safety report		
	Communication	62 (14%)
	Employee behavior	21 (5%)
	Environment	28 (6%)
	Equipment	65 (15%)
	Issue related to patient assessment	19 (4%)
	Issues related to resident and staff training	114 (26%)
	Lack or misinterpretation of info	32 (7%)
	Nursing documentation	8 (2%)
	Patient or family behavior	24 (5%)
	Policies and procedures	49 (11%)
	Safety and security	11 (2%)
	Supplies	8 (2%)
Type of education		
	No education required	159 (31%)
	Directed feedback	235 (46%)
	Quarterly/monthly update	100 (20%)
	Urgent communication	15 (3%)
	Provider simulation	2 (<1%)

**Table 2:** Patient safety note (PSN) issues.

PSN Issue	% (N)	Example
Issues related to resident and staff training	25% (129)	Sharps left at bedside after a procedure
Communication	18% (93)	Consultant recommendation delay
Equipment	14% (71)	Limited accessibility to end tidal CO_2_ in all rooms of ED
Policies and procedures	13% (69)	Provider questioning the process that led to a patient with a positive pregnancy test having imaging done
Lack or misinterpretation of info	9% (44)	Patient arrived after treatment from an outside area on antibiotics that were not effective for the infection he had
Employee behavior	9% (47)	Provider noted to enter a droplet isolation room without proper PPE
Issue related to patient assessment	7% (34)	Patient treated for gout and was later found to have osteomyelitis
Environment	6% (43)	Bedbug found in a patient care location
Patient or family behavior	5% (27)	Patient elopement
Safety and security	4% (21)	Assault by patient with security and police response
Documentation	3% (15)	Assessment found in wrong patient’s chart
Supplies	2% (9)	Myelogram kit was supplied in place of standard lumbar puncture kit and this had three specimen vials instead of the expected four

66 PSNs noted multiple issues: 2 with four issues, 11 with three issues, and 55 with two issues.

*ED*, emergency department, *CO_2_*, carbon dioxide; *PPE*, personal protective equipment.
